# 182. Impact of Rapid Identification and Phenotypic Results on Clinical, Economic, Antimicrobial Stewardship Outcomes in Patients with Gram-Negative Bacteremia

**DOI:** 10.1093/ofid/ofac492.260

**Published:** 2022-12-15

**Authors:** Harold Shoop, Amy Fabian, Shaina Doyen

**Affiliations:** Baptist Health Louisville, Louisville, Kentucky; Baptist Health Louisville, Louisville, Kentucky; Baptist Health System, Louisville, Kentucky

## Abstract

**Background:**

Timely and effective antibiotic therapy is a critical determinant of clinical outcome in Gram-negative bacilli (GNB) bloodstream infections (BSI). Conventional antimicrobial susceptibility testing (AST) remains a rate-limiting step in determining targeted therapy. Accelerate Pheno^™^ (AP) is a novel system providing both identification (ID) and AST results within 8 hours. The primary objective of this study was to determine the impact of rapid ID and AST with AP on time to achieving targeted therapy (TTT).

**Methods:**

This single center, quasi-experimental study compared outcomes with AP (POST; November 2020 to July 2021) versus a historical standard (PRE; November 2019 to July 2020) of rapid diagnostic testing (BioFire® FilmArray®BCID) and conventional AST (VITEK®2) with pharmacist-driven antimicrobial stewardship (AMS) intervention. PRE and POST BSI were retrospectively identified by positive blood culture (BCx) with an on-panel GNB. Inpatients were excluded if less than 18 years of age, pregnant, polymicrobial BCx, BCx with the same organism and source within 7 days, or if died or transitioned to palliative or hospice care before final AST results. Secondary outcomes included inpatient all-cause mortality, hospital and ICU length of stay, hospital-acquired *Clostridioides difficile* infection, antibiotic and microbiologic costs, days of anti-pseudomonal therapy, antibiotic intensity score, and time to first intervention following Gram stain, ID, and AST results.

**Results:**

Overall, 373 BSI were screened and 294 were included [161 PRE vs 133 POST]. Baseline demographics and characteristics were similar between groups. Median TTT was shorter POST [50 hours (IQR 44–92) vs 28 hours (IQR 14–55), p < 0.05]. Median microbiology testing costs per episode were higher POST [Δ$40.33 (32%), p < 0.05]. Other secondary outcomes did not differ significantly between groups. Pharmacists intervened more POST [69 (43%) vs 77 (58%), p < 0.05]. AP completed ID and AST in 155 of 205 (76%) BSI. Off-panel GNB occurred in 26 (13%) BSI POST.

**Conclusion:**

Implementing the AP system together with an established AMS program resulted in a shorter TTT and a higher cost to test. Clinical, AMS, and other economic outcomes were not impacted by the addition of AP.

**Disclosures:**

**All Authors**: No reported disclosures.

